# Impact of patient frailty on outcome in cardiothoracic surgery

**DOI:** 10.1186/cc14276

**Published:** 2015-03-16

**Authors:** J Brohan, P Delaney, B O Brien

**Affiliations:** 1 Cork University Hospital, Cork, Ireland

## Introduction

Frailty is defined as a multidimensional syndrome involving loss of physical and cognitive reserve leading to greater vulnerability to adverse events [1]. Such events include susceptibility to unplanned hospital admissions, and death [1-3]. Frailty is associated with increased ICU and 6-month mortality, and reduced quality of life [4]. The aim of this study is to investigate the impact of baseline frailty on postoperative quality of life indicators and postoperative frailty following cardiothoracic surgery.

## Methods

Adult patients undergoing cardiac surgery or thoracic surgery (involving thoracotomy) were included in this study. Baseline measures of frailty [4] and performance status were prospectively recorded using validated tools. Informed consent was obtained prior to inclusion. Outcome measures of APACHE II scores, duration of ventilation, length of ICU stay and mortality were recorded. Follow-up at 6 months was conducted by telephone to assess recovery patterns.

## Results

A total of 120 patients were included in this study, including 100 patients who underwent cardiac surgery and 20 patients who underwent thoracic surgery. Eighty-five patients (70.8%) were male. The mean age was 65.4 years (range 25 to 89 years). The mean baseline frailty score also varied widely within our cohort. Four patients died in the ICU following their surgery (3% ICU mortality rate). Mean length of ICU stay was 2.7 days (range 0 to 20 days), with a mean duration of ventilation of 20 hours (range 0 to 264 hours). Follow-up of these patients at 6 months following their surgery is currently underway.

## Conclusion

Due to advances in life expectancy, health and perioperative medicine, it has become more difficult to determine fitness for major surgery. Our data suggest that frailty may be a useful prognostic measure to help inform such decisions.
